# Complete Genome Sequence of a Nephropathogenic Infectious Bronchitis Virus Strain Isolated in China

**DOI:** 10.1128/genomeA.00815-13

**Published:** 2013-10-10

**Authors:** Jing-tian Yang, Bing-cun Ma

**Affiliations:** Ecological Security and Protection Key Laboratory of Sichuan Province, Mianyang Normal University, Mianyang, China

## Abstract

Infectious bronchitis virus (IBV) causes tremendous economic losses to the poultry industry. Here, we report the complete genome analysis results for a new natural recombination nephropathogenic IBV strain named SAIBK, which was isolated in the Sichuan province of China in 2005.

## GENOME ANNOUNCEMENT

Infectious bronchitis virus (IBV), a pathogen causing highly contagious and acute disease in domestic chickens, belongs to group III of the genus *Coronavirus* in the family *Coronaviridae* (1). It is an enveloped, unsegmented, positive-sense, single-stranded RNA (ssRNA) virus and has a genome of approximately 27.6 kb (2). Recently, many epidemiological analysis reports have suggested that nephropathogenic IBVs have become increasingly prevalent (3–6) in China. In this work, the complete genome sequence of an isolate named SAIBK was analyzed and recombination was detected between SAIBK and some previously reported IBVs.

A rapid amplification of cDNA ends (RACE) kit (TaKaRa, Japan) was used to obtain the 5′ and 3′ ends of the genome. Other parts were amplified by 19 primers with overlap between each fragment and were cloned into the pMD19-T vector (TaKaRa, Japan). All fragments were sequenced three times by Sangon Biotech (Shanghai, China). The sequenced fragments were assembled using the SeqMan software program (DNAStar, Inc.). Sequence alignment was conducted and a phylogenetic tree was constructed using the software program MEGA5 (7). Recombination analysis was performed using the RDP 4.14 (8) and SimPlot 3.5.1 (9) software programs.

The complete genome of the SAIBK strain is 27,534 nucleotides (nt) in length, including the poly(A) tail. It has a classical IBV genome organization with 10 open reading frames (ORFs): 5′-1a-1b-S-3a-3b-E-M-5a-5b-N-3′. The genome sequence of SAIBK shows the highest identity (94.3%) to the Chinese IBV strain SC021202 (GenBank accession no. EU714029) and the lowest identity (85.8%) to two Chinese IBV strains, BJ (GenBank accession no. AY319651) and DY07 (GenBank accession no. HM245923). It has lower nucleotide identities of 88.1%, 87.9%, and 87.7% to the most popularly used IBV vaccine strains, H120, H52, and M41, respectively.

Phylogenetic analysis of the complete genome results indicated that the SAIBK strain clusters into the same branch as the IBV YN strain (GenBank accession no. JF893452) and the SC021202 strain (GenBank accession no. EU714029). The S1 subunit of the IBV genome is the major determinant of serotype (10–13), and S1 analysis indicated that the SAIBK strain has a 4/91-like serotype.

The employed recombination detection methods revealed that SAIBK is a chimera virus, with recombination by the SC021202 strain as a major parent and the H120 vaccine strain as a minor parent. The first and second recombination regions were located at positions 7231 to 9126 and 13437 to 14473 in genes 1a and 1b, respectively. There were two other recombination regions detected at positions 951 to 1067 and 5393 to 5605 of SAIBK, which were recombined with the SC021202 strain as a major parent and the H52 vaccine strain as a minor parent. The recombination detection results suggested that SAIBK is possibly a chimera virus derived from the popularly used vaccine strains H120 and H52 and the field strain SC021202, and the SC021202 strain was isolated from chickens vaccinated with H120 in the Sichuan province of China in 2003 (14). This result revealed that the field IBVs in Sichuan Province have undergone genetic recombination and are possibly emerging as new mutant strains, such as SAIBK.

### Nucleotide sequence accession number.

The complete genome sequence of the SAIBK isolate was submitted to GenBank and assigned the accession no. DQ288927.
